# Predictive factors of hospital length of stay in patients with operatively treated ankle fractures

**DOI:** 10.1007/s10195-013-0280-9

**Published:** 2013-12-14

**Authors:** Matthew R. McDonald, Vasanth Sathiyakumar, Jordan C. Apfeld, Benjamin Hooe, Jesse Ehrenfeld, William T. Obremskey, Manish K. Sethi

**Affiliations:** The Vanderbilt Orthopaedic Institute Center for Health Policy, Vanderbilt University, Suite 4200, South Tower, MCE, Nashville, TN 37221 USA

**Keywords:** Ankle fracture, Postoperative length of stay, ASA score, Payment and reimbursement model, Surgical outcomes, Healthcare costs

## Abstract

**Background:**

Operative fixation of ankle fractures is common. However, as reimbursement plans evolve with the potential for bundled payments, it is critical that orthopedic surgeons better understand factors influencing the postoperative length of stay (LOS) in patients undergoing these procedures to negotiate appropriate reimbursement. We sought to identify factors influencing the postoperative LOS in patients with operatively treated ankle fractures.

**Materials and methods:**

Six hundred twenty-two patients with ankle fractures between January 1st, 2004 and December 31st, 2010 were identified retrospectively. Charts were reviewed for gender, length of operative procedure, method of fixation, American Society of Anesthesiologists (ASA) physical status score, medical comorbidities, and postoperative LOS. Both univariate and multivariate models were developed to determine predictors of patient LOS. Financial data for an average 24-h inpatient stay were obtained from financial services.

**Results:**

Six hundred twenty-two patients were included. In a linear regression analysis, a statistically significant relationship was demonstrated between ASA status and LOS (*P* < 0.001). Multiple regression analysis further characterized the relationship between ASA and LOS: a 1-U increase in ASA classification conferred a 3.42-day increase in LOS on average (*P* < 0.001). Based on an average per-day inpatient cost of $4,503, each unit increase in ASA status led to a $15,490 increase in cost.

**Conclusions:**

Our study demonstrates that ASA status is a powerful predictor of LOS in patients undergoing operative fixation of ankle fractures. More complete understanding of these factors will lead to better risk adjustment models for measuring outcomes, determining fair reimbursement, and potential improvements to the efficiency of patient care.

**Level of Evidence:**

Level III retrospective comparative study regressing length of stay with many variables, including ASA physical status.

**Electronic supplementary material:**

The online version of this article (doi:10.1007/s10195-013-0280-9) contains supplementary material, which is available to authorized users.

## Introduction

Musculoskeletal injuries have become increasingly more common in the USA, with approximately three out of every five injuries occurring to this system [1]. Among these injuries, sprains/strains represent the most frequent (44 %), and fractures represent the second most frequent (25 %) [1]. Between 2006 and 2007, an estimated 16.2 million outpatient and inpatient fractures were treated [1]. One population-based study reports that ankle fractures occur in 187 per 100,000 persons every year [4]. The CDC estimated that, in 2003 alone, over 1.8 million people visited the emergency room because of ankle and lower leg injuries [9].

A prospective cost analysis study of 30 patients with unstable ankle fractures found the total inpatient hospital cost to be $1,801 per patient and the total outpatient cost to be $333 per patient [2]. Given the prevalence of these operations, it is imperative to seek ways to reduce these costs. Healthcare costs continue to be a central issue in the US economy, especially with the signing of healthcare reform legislation in 2010. With impending changes such as the transition to a bundled payment system, it is crucial for orthopedic trauma surgeons to develop a better understanding of cost drivers for the treatment of ankle fractures.

One avenue of interest in the pursuit of such variables is the role of the American Society of Anesthesiologists (ASA) classification score, which is based on the anesthesiologist’s evaluation of the patient’s health status and comorbidities prior to an operation [7]. The ASA score has been proven to be an effective predictor of patients with an increased risk of complications, including perioperative risk assessment, perioperative mortality, as well as postoperative outcome [10–12], with higher scores associated with increased risk of complications. ASA scores have also been shown to be a significant predictor in length of stay (LOS) and therefore cost management of patients undergoing operative fixation of hip fractures [5] as well as return-to-function status [3]. Therefore, this retrospective study sought to elucidate the relationship between various patient variables, such as ASA score, length of operative procedure, method of fixation, medical comorbidities, and postoperative LOS, in patients undergoing open treatment of an ankle fracture.

## Materials and Methods

After obtaining approval from our institutional review board, all patients who underwent open treatment of ankle fractures between January 1, 2004 and December 31, 2010 were identified through a current procedural terminology (CPT) code search (Appendix Supplementary Table 1). The following metrics were extracted from the selected patients’ records: date of birth, height, weight, date of admission, age at time of procedure, start and stop times of procedure, duration of procedure, and whether or not procedure was an emergency. Other data acquired from the patients’ charts were total LOS, days from admission to surgery, days from surgery to discharge, and any documented complications. In addition, a history of other medical comorbidities for each patient was acquired, including prior myocardial infarction (MI), dysrhythmia, atrial fibrillation, congestive heart failure (CHF), heart block, cerebrovascular disease, chronic obstructive pulmonary disease (COPD), emphysema, current smoking status, prior smoking history, renal disease, dialysis, cancer, and diabetes. No patient identifiers were included in this deidentified database. In addition, ASA classification was also obtained for each patient. This value was assigned to each patient by the anesthesiologist just prior to the start of the operative procedure. Incomplete charts were excluded from the analysis.

The average total cost to the hospital of an inpatient day at the institution was obtained from hospital financial services, and the average cost of an inpatient day was treated as a unit cost per inpatient day. The length of stay for each patient was multiplied by this unit cost to estimate the cost of inpatient postoperative care per patient for a given visit. Both univariate and multivariate models were developed to determine predictors of length of stay and thus cost of the length of stay postoperatively.

## Results

After exclusion of incomplete charts, 622 patient charts were available for analysis. Basic demographic information is provided in Table 1. The average patient age was 44.58 years, and the average LOS was 5.59 days. All patients were ASA class 1 through 4, with the majority of patients being class 2.

**Table 1:** Population demographic information

	*N*	%
Age (years)		
0–9	0	0.00
10–19	46	7.40
20–29	116	18.65
30–39	99	15.92
40–49	104	16.72
50–59	114	18.33
60–69	77	12.38
70–79	42	6.75
80–89	16	2.57
90–99	1	0.16
>99	7	1.13
Gender		
Female	305	49.04
Male	317	50.96
Race		
Caucasian	497	79.90
African American	77	12.38
Hispanic	19	3.05
Asian	2	0.32
American Indian	1	0.16
Other/unknown	26	4.18
ASA status		
1	57	9.16
2	328	52.73
3	196	31.51
4	41	6.59
Other		
Mean length of stay (days)	5.59	
Mean duration of surgery (min)	137.11	

In a linear regression analysis, a statistically significant relationship was demonstrated between ASA score and length of stay (*P* < 0.001). Multiple regression analysis was conducted to further characterize the relationship between ASA score and LOS: a 1-U increase in ASA score conferred a 3.42-day increase in LOS on average (*P* < 0.0001). The average total cost to the hospital of an inpatient day at the institution was found to be $4,530. Treating the average inpatient daily cost as a unit cost, a 1-U increase in ASA led to a $15,490 increase in cost to the institution. Table 2 summarizes the multivariate regression of comorbidities on LOS. No single comorbidity reached statistical significance as a predictor of LOS.

**Table 2:** Results of regression analysis of comorbidities on LOS

Comorbid condition	LOS	Cost^a^	*P* value
MI	0.64565	$2,924.79	0.7829
Dysrhythmia	0.54559	$2,471.52	0.7704
Hypertension	−0.00485	−$21.97	0.9968
CHF	2.10027	$9,514.22	0.4892
Heart block	1.42492	$6,454.89	0.7458
Renal insufficiency	0.55606	$2,518.95	0.8498
Dialysis	3.02694	$13,712.04	0.6605
Diabetes	−1.31132	−$5,940.28	0.4759
Cancer	−1.7441	−$7,900.77	0.3589
Cocaine use	−1.30578	−$5,915.18	0.7092
Alcohol use	−2.71025	−$12,277.43	0.1523
Opiate use	2.58596	$11,714.40	0.6979

^a^Cost is a direct multiplication of average per diem inpatient cost by LOS

Table 3 describes the length of stay data for each ASA classification 1 through 4. A predicted total cost to the institution for the inpatient stay of a patient with ASA score 1 through 4 was calculated utilizing the data provided by financial services for the cost of a single-day inpatient stay. A relatively small difference in mean LOS between ASA score of 1 and ASA score of 2 was observed, as would be expected as both of these groups represent generally healthy patients. However, a steep rise in the mean LOS from 3.84 to 8.07 days was observed between ASA scores 2 and 3, respectively.

**Table 3:** Comparison of ASA physical status, mean LOS, mean operative duration, predicted costs, and actual costs

ASA status	Patients	Mean LOS (days)	Mean operative duration (min)	Predicted cost	Actual cost
1	57	2.58	127.37	$3,418	$11,687
2	328	3.84	134.64	$19,536	$17,395
3	196	8.07	147.29	$36,118	$36,557
4	41	11.95	121.88	$49,423	$54,134

**Fig. 1:**
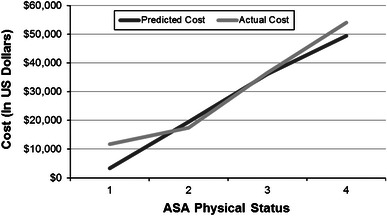
Relationship between ASA physical status and both mean predicted costs and mean actual costs

Figure 1 depicts the trend in the actual average cost of postsurgery hospitalization for an ankle fracture patient based on the ASA classification.

## Discussion

Our data show that a 1-U increase in the ASA score of patients undergoing operative repair of an ankle fracture is associated with a 3.42-day increase in average LOS. These results complement those previously published regarding the positive association between ASA physical status and average LOS in hip fracture patients, confirming that the ASA physical status classification system is a powerful predictor of LOS. Given that a particular patient’s ASA physical status can seldom be altered prior to surgery, the utility of this information is more beneficial in terms of budgeting and planning for patients undergoing open treatment of ankle fractures. ASA physical status and daily costs are commonly collected, which makes tracking of these data for real-time budgeting and bed management easily employable at essentially any institution. Furthermore, as reimbursement systems change from a fee-for-service model to a fixed-reimbursement model, this study highlights the utility of a tiered reimbursement model for each diagnosis based on relatively static patient factors such as ASA physical status.

While LOS was the only covariate found to be predictive of LOS, one of the limitations of this study is that not all possible patient variables could be accounted for, and thus it remains possible that some other single factor not included in this analysis better predicts LOS variation rather than ASA physical status. Furthermore, an intricate interplay undoubtedly exists between multiple comorbidities and patient variables that predict LOS. ASA physical status was designed expressly for the purpose of integrating all of a patient’s comorbidities into a single value, and this is why the authors believe it is such a good predictor of LOS in orthopedic trauma patients.

In conclusion, this study demonstrates the powerful nature of the ASA classification for explanation of variance of length of stay for hospitalized patients. This method can easily be integrated into almost any hospital, since ASA and costs are universally collected. This model may be utilized to predict a patient’s postoperative course more accurately, which has benefits for predicting costs, budgeting, and management of patient beds. Additional research may also be done to investigate associations between ASA physical status and other cost drivers in addition to LOS.

Other limitations to this study include the fact that this particular patient population was from a single, tertiary medical center, which may receive “sicker” patients, and therefore may not be indicative of the general population. Other factors such as surgeon, surgical technique, delayed discharge, pain control, or insurance status could not be included in the multivariate regression and may all also contribute to LOS. Finally, one of the major limitations of utilizing ASA score is the fact that research has shown it to have little to moderate interrater reliability [6, 8].

## Electronic supplementary material

Below is the link to the electronic supplementary material.

Supplementary material 1 (DOCX 13 kb)
