# Supplementary material for: Predictive factors of hospital length of stay in patients with operatively treated ankle fractures
Source: J Orthop Traumatol. 2013 Dec 14;15(4):255–8. doi: 10.1007/s10195-013-0280-9 (PMC4244567; doi:10.1007/s10195-013-0280-9)
Supplement: Supplementary file 1 — Supplementary material 1 (DOCX 13 kb) [file 10195_2013_280_MOESM1_ESM.docx]

| **Supplementary Table 1.** Ankle Fracture CPT codes | |
| --- | --- |
| **CPT Code** | **Description** |
| 27766 | OPEN TX MEDIAL MALLEOLUS FX W/WO INT/EXT FIXA |
| 27784 | OPEN TX PROX FIBULA/SHAFT FX W/WO INT/EXT FIXA |
| 27792 | OPEN TX DISTAL FIBULAR FX W/WO INT/EXT FIXA |
| 27814 | OPEN TX BIMALLEOLAR ANK FX W/WO INT/EXT FIXA |
| 27822 | OPEN TX TRIMALLEOLR FX MED/LAT; WO FIXA POST L |
| 27823 | OPEN TX TRIMALLEOLAR FX MED/LAT; W/FIXA POST L |
| 27829 | OPEN TX DIST TIBIOFIBULAR JT DISRUPT W/WO FIXA |
